# Associated Factors of Postpartum Modern Contraceptive Use in Burie District, Amhara Region, Ethiopia

**DOI:** 10.1155/2020/6174504

**Published:** 2020-03-21

**Authors:** Wassachew Ashebir, Tilahun Tadesse

**Affiliations:** ^1^College of Health Science, Debre Markos University, Ethiopia; ^2^East Gojam Zone Health Office, Ethiopia

## Abstract

**Methods:**

A community-based cross-sectional study was employed among 686 mothers in Burie District from March 16 to March 25, 2017. A multistage sampling technique was used to select the study participants. Data were collected using face to face interviewer administered structured questionnaires. Then, the collected data was entered, coded, and cleaned into EPI Data version 3.1 and exported to SPSS version 20.0 for data analysis. Bivariate and multivariate logistic regression was done to assess the association of factors with postpartum modern contraceptive use. Adjusted odds ratios with 95% confidence intervals were calculated, and *p* values <0.05 were considered to indicate statistical significance.

**Result:**

This study revealed that postpartum modern contraceptive use was found to be 20.7%. Postpartum modern contraceptive use was significantly associated with women's level of education (AOR = 0.15, 95% CI (0.03-0.71)), discussing FP methods with partner (AOR = 0.60, 95% CI (0.40-0.90)), knowing menses return after birth (AOR = 0.39, 95% CI (0.25-0.59)), ever heard about modern FP methods (AOR = 0.06, 95% CI (0.01-0.43)), and contacting health professionals (AOR = 1.85, 95% CI (1.19-2.88)). *Conclusion and Recommendations*. Postpartum modern contraceptive use was found to be low. Therefore, health professionals should work on improvements in women's educational status, making awareness of the women and counseling of their husbands about the use of postpartum contraception, when fertility returned and risky timing for becoming pregnant.

## 1. Introduction

Pregnancies occurring within a year of the mother's previous birth are highly at risk for the health of both the mother and the child than those occurring later [1]. This is because closely spaced pregnancies are associated with higher risks of abortions, bleedings, anemia, and poor pregnancy outcomes like preterm babies, small for gestational age babies. In spite of this fact, the first 12 months after giving birth in relation to family planning (FP) use is the time which is often given less attention by health care managers, health service providers, and users. Even, most women do not realize that they are at risk for subsequent pregnancy. Due to this, there is an increased substantial risk of unwanted conception and an often-frustrated desire for contraceptive protection [2]. As a result, many women in the postpartum period did not start use of any modern contraceptive method. From 7 to 9 months after birth, most women become exposed to pregnancy but do not want to become pregnant again so soon, yet still do not obtain contraceptive protection. Such women have experienced a return of menses, are not abstaining from intercourse, and are unprotected from conception [3, 4].

Modern family planning is an important method that saves lives of women and children and improves the quality of life for both women and children. It is one of the best investments which help to ensure the health and well-being of women, children, and communities [5]. Initiation of family planning use during the postpartum period is most critical to improve maternal and child health. By definition, postpartum family planning is defined as the prevention of unintended pregnancy and closely spaced pregnancies through the first 12 months after a child birth. In this regard, World Health Organization (WHO) recommends Postpartum Family Planning (PPFP) as a critical component of health care that has the potential to meet women's desire for contraception and save millions of maternal and infant lives in low- and middle-income countries [6].

The postpartum period is an essential period for addressing the greater unmet needs in family planning and for reducing the risks of closely spaced pregnancies. However, during this period, most women do not recognize that they are at risk for pregnancy. As a result, many of them fail to start use of any modern contraceptive method. Hence, addressing the unmet need for family planning during the postpartum period is critical in maternal and child health. Many studies have shown that family planning use within a year following birth, and child and maternal mortality risk are inversely related. As birth interval reduced, neonatal and child mortalities increased [7]. Birth spacing of at least 2 years is considered essential for maintenance of good health of women and their children during and after childbirth or breastfeeding. In such sense, ensuring that births are adequately spaced is linked to early use of an effective family planning method [6].

However, in Ethiopia, evidence has been shown that nearly half (47%) of all pregnancies occur within a short birth interval of less than two years after the preceding birth [8]. Moreover, the country is characterized by a very high fertility, high maternal and child mortality, and high infant mortality [9, 10]. On the opposite, the use of postpartum family planning could greatly reduce maternal mortality and morbidity by 75%, reduce unwanted and mistimed pregnancies by two-thirds, and reduce the risks of abortion by 73% [11, 12].

Efforts like training health workers on FP, increasing health institutional delivery, increasing postnatal care service, and establishing health posts with assigning 2-3 health extension workers in each kebeles had been made by the Ethiopian government through the Ministry of Health and other stakeholders to reduce the unmet need for family planning among the postpartum women. Despite these efforts, the unmet need for postpartum family planning in Ethiopia is still high, ranging from 86% in the first 5 months to 76% by the end of first year after delivery [13, 14]. In addition, some policy makers and program managers showed their uncertainty about incorporating PPFP into their unique national and local contexts, especially in areas with cultural barriers to family planning for postpartum women and with low facility-based delivery coverage. Others misunderstood or underestimated the risk of pregnancy in the postpartum period and believed that PPFP was either unnecessary or a less important investment than family planning for nonpostpartum women [15].

As a study done in North West Ethiopia, 10% of postpartum women within 12 months were using a contraceptive method mostly for spacing children for which, injectable was the most popular method [16].Another study conducted in Kiramu District, Western Ethiopia indicated that only 28.3% women used different types of contraceptives during the postpartum period [17].

A number of factors were attributed for non-use of modern FP in the postpartum period: limited knowledge on available choice, failure to integrate PPFP counseling with ANC, delivery and PNC services, confusion as to when fertility comes back and unpredictability of the timing of the onset of intercourse, myths and misconceptions about PPFP, and lack of standardized protocol for PPFP service delivery [18]. Hence, this study had addressed this problem by providing special attention about modern F/P method utilization in women of reproductive age within one year after giving birth.

## 2. Methods

### 2.1. Study Setting, Population, and Design

A community-based cross-sectional study was conducted in Burie District from March 16 to March 25, 2017. Burie District is located at 411 kilometers to the North West of Addis Ababa, the capital city of Ethiopia, and 165 km from Bahir Dar, the capital city of Amhara National Regional State. According to the Burie District administration health office report, the current (2017) population of the District was estimated at 128,320, from these, 25,959 were women in reproductive age (15-49 years) who were expected to take any method of family planning service and 4,324 (3.37%) women who were expected to become pregnant. There are twenty kebeles (19 rural and 1 urban kebeles) in the District. There are five governmental health centers, three lower private clinics, both of them providing family planning services. According to the 2008 E.C annual report, the total family planning coverage of Burie District was 82.91%, but the data about modern family planning utilization among women in reproductive age group within one year of live birth was not well organized and studied. The source population comprised of all reproductive-age women (15-49 years) who gave live birth within one year in Burie District.

### 2.2. Sample Size and Sampling Procedure

The sample size was calculated using the single population proportion formula *n* = (Z *α*/2)2 P(1P)/(d2), considering the 28.3% prevalence of postpartum modern contraceptive use in Kiramu District, Western Ethiopia [17], the use of a 95% confidence interval (CI), design effect of 2, and a 5% margin of error (d). After adding a 10% nonresponse, the total sample size was calculated to be 686. A multistage sampling technique was used to select participants in the study area. The total 20 kebeles in the district were classified in to 19 rural and one urban kebeles by stratified sampling technique. Then, seven kebeles (six rural kebeles with simple random sampling or lottery method and one urban kebele purposively) were selected. The list of mothers who gave live birth within one year in each selected kebele was obtained from the registration of the health post in the kebeles and served as a sampling frame. The total calculated sample size was proportionally distributed to each selected kebele based on the number of women who gave live birth within one year living in each selected kebele. Finally, proportionally allocated respondents to each selected study kebeles was randomly selected through computer-generated numbering technique.

### 2.3. Data Collection

A structured and pretested questionnaire was prepared first in English and translated to the local language (Amharic) and translated back to English in order to assess its consistency. Data were collected by seven women diploma holder midwives and one BSc nurse for supervision. Face-to-face interviews were conducted to collect the data. Data quality was checked during questionnaire designing, data collection, and data entry. The collection questionnaire was pretested among 5% of study subjects to none selected kebele of Burie District. The data collectors and supervisors were trained at district town (Burie) for one day on the objectives of the study and data quality The questionnaire was adopted from previous studies and was modified according to the context of the study area [19, 20].

### 2.4. Data Processing and Analysis

All returned questionnaires were checked manually for the completeness and consistency of responses. The collected data were coded and entered in EpiData version 3.1 and exported to SPSS version 20.0 for analysis. Descriptive statistics were computed for each study variables. Both bivariate and multivariate logistic regressions were used to identify factors associated with postpartum modern contraceptive use. Variables with a *p* value <0.2 in the bivariate analysis were entered into a multivariate analysis to identify independently associated significant variables of postpartum modern contraceptive use. Adjusted odds ratios (AORs) with 95% CIs were used to identify factors associated with postpartum modern contraceptive use. The *p* values less <0.05 were considered to indicate statistically significant of the associations with postpartum modern contraceptive use.

### 2.5. Ethical Considerations

Ethical clearance was obtained from the ethical review committee of the College of Health Science, Debre Markos University, and communication was done with Burie District Health Office before the study take place to build trust with local kebeles and study communities during data collection time. Informed consent was obtained from the respondents, because participants should be informed that they have the full right to withdraw, reject, or stop immediately at any time from the interview if they have no willingness to participate in the study. Personal identification was not used in the questionnaire, and confidentiality was assured throughout the study period.

## 3. Result

### 3.1. Sociodemographic Characteristics of Respondents

Overall, 681 (97.3%) postpartum women participated in to the interview process. The age range of the respondents was 18-46 years. The women's mean ± SD age was 30.26 + 5.492 SD years, and 227 (33.3%) were aged between 25 and 29 years. The majority (94.1%) of the respondents were married. The majority (98.4%) were Amhara by ethnicity. Of the respondents, 671 (98.5%) were Orthodox Christians. Regarding occupational status, majority of the respondents (83.8%) were farmers, 375 (55.1%) had no media access, 405 (59.5) of women and 364 (56.78%) of husbands cannot read and write. Four hundred thirty-seven (64.2%) of the respondents had monthly income of <1,000 ETB (Table 1).

### 3.2. Reproductive Characteristics of Respondents

This study revealed that women married for the first time as early as 10 years and as late as 28 years of age. The mean age of respondents during their first marriage was 16.17 SD + 3.416, and the median age was 16 years, whereas the mean age of respondents during their first child birth was 19.97 SD + 3.38, and the median age was 20 years with a minimum and maximum age of 15 and 35 years. Majority of the respondents, women during their first marriage and first child birth were found in the age group of 15-19 years with 350 (51.4%) and 331 (48.6%), respectively. The rest 220 (23.3%) and 111 (16.3%) of women got their first marriage at the age group of 10-14 and 20-24 years, respectively, and 289 (42.5%) and 58 (8.55) of women got their first birth at the age group of 20-24 and 25-29 years, respectively, but only 3 (0.4%) got their first birth at the age group of >30 years.

Most respondents, 304 (44.6%) had 1-2 pregnancies, and the average number of birth was 1.72 (SD ± 0.794). About 317 (37.5%) of women gave 1-2 children followed by 256 (37.6%) who gave 3-4 children, and the average number of alive children were 1.7 (SD ± 0.774). More than half women (385 (56.5%)) had a desire of 4-5 children, and 191 (28%) wanted to have 6-7 children (Mean value of 3.18 ± 0.774 SD) through their life. Among the respondents, 49 (7.2%) of them had a history of pregnancy within one year before the index birth due to husband disapproval to use modern FP methods. Of the study participants, 67 (9.8%) were pregnant after the index birth, and 54 (80.6%) pregnancies occurred within 9-12 months after the index birth (Table 2).

### 3.3. Maternal Health Services Use-Related Characteristics of the Study Participants

Five hundred ninety-seven (87.7%) women had visited health institutions and counseled about modern FP methods. One hundred seventy-four (25.6%) respondents got counseling during PNC, 159 (23.3%) during ANC, 129 (18.9%) during delivery, and 136 (20%) at any health service visits. Depo-Provera (36.7%) and Implanon (30.5%) were the commonly counseled FP methods. Two hundred thirteen (35.73%) and 207 (34.73%) women were counseled about FP side effect and route of administration. Majority (60.74%) of women responded as waiting from 10 to 20 minutes to get the service in health institution. Two hundred eighty-seven (42.1%) of mothers preferred health posts for their FP service, 197 (28.9%) preferred health centers, and 29 (4.3%) preferred private pharmacy/drug vendors. Four hundred forty three (65.1%) and 376 (55.2%) of women agreed about good and respectful approach of health professionals and regular service provision in FP class room, respectively.

The study showed that majority (60.1%) of respondents did not know about the return of menses. Of those who knew about the return of menses after birth, 125 (46.5%) responded that the time of menses return was less than six months. Two hundred eighty-seven (42.1%) of the respondents knew about the occurrence of pregnancy without the return of menses after birth, and most of them (54.86%) got information from HEWs followed, but 394 (57.9%) of respondents did not know about it. Women who had a history of abortion were 56 (8.2%). From these, 30 (53.57%) were unplanned, and 26 (46.43%) were unwanted pregnancy. The most common causes of abortion were unplanned pregnancy (53.57%) in which 66.03% of it was terminated by medical induced abortion.

Among the respondents interviewed, 596 (87.5%) heard about modern family planning methods, and most of them (44.63%) got information from health extension workers followed by from their friends (22.48%). From those who got information about modern FP methods, 288 (38.3%) of them heard about Depo-Provera followed by Implanon (29.7%). Four hundred thirty-five (72.98%) respondents heard that modern FP has an advantage of spacing child birth, and sixteen respondents heard more than one advantage of modern FP methods. Modern family planning method was discussed as an issue in 267 (39.2%) of the respondents with their partners, and in 146 (54.5%) of them, decision was made by both of them after discussion.

### 3.4. Postpartum Modern Contraceptive Use of Respondents

The finding of this study showed that 141 (20.7%) of mothers had utilized modern family planning methods within one year of live birth while 540 (79.3%) did not. From respondents using modern FP method after the index birth, 85 (60.35) of them started within forty-five days to three months after birth. Among modern FP methods used currently, Depo-Provera were more utilized (48.93%) followed by Implanon (28.4%). Jadelle were more likely used before the index birth than current users (7 : 1), and condom was not totally used by any of the mothers.

Five hundred forty (79.3%) respondents mentioned about the main reasons for not taking modern FP methods. Among the reasons were as follows: menses not returned (started) to use FP methods (27.59%), fear of side effects (18.33%), no prior exposure to modern FP (13.33%), desire for more children (13.33%), and husband disapproval (10.93%). From those side effects experienced by women, heavy vaginal bleeding (70.71%) was followed by severe headache (15.15%).

### 3.5. Factors Affecting Postpartum Modern Contraceptive Use of Respondents

In the multivariate logistic regression analysis, the following five variables were identified as independently associated with postpartum modern contraceptive use. These were educational status, discussion with partner about modern FP, menses returning after birth, ever heard about modern FP methods, and availability of health professionals to provide FP service.

Women who attended primary school were 15% more likely to report postpartum modern contraceptive use than those who cannot read and write (AOR = 0.15; 95% CI, 0.03-0.71).

Women who discuss modern FP with partner were 60% more likely to report postpartum modern contraceptive use than those who did not (AOR = 0.60; 95% CI, 0.40-0.90). The odds of using modern contraceptive use for women with returned menses was 39% higher compared to those not returned menses (AOR = 0.39; 95% CI, 0.25-0.59). Women who ever heard about modern FP methods were 6% more likely to report postpartum modern contraceptive use than those who did not (AOR = 0.06; 95% CI, 0. 01-0.43). The odds of using modern contraceptive use for women contacting health professionals was 1.85 times higher compared to those who did not get health professionals that provide FP service (AOR = 1.85; 95% CI, 1.19-2.88) (Table 3).

## 4. Discussion

This study was conducted to assess postpartum modern contraceptive use and associated factors among women within one year of live birth in Burie District, Ethiopia. Nearly one-fifth (20.7%)) of the women reported using modern contraceptive methods during the postpartum period.

Postpartum modern contraceptive use was significantly associated with women's educational level, discussing modern family planning with partner, menses returning since birth, ever heard about modern FP, and having contacted health professionals providing the service.

The finding of this study revealed that 141 (20.7%) of women utilized modern FP during the postpartum period. This is lower than the study carried out in Kiramu District, Western Ethiopia (28.3% [17], and two low-income countries—Zambia and Kenya (46%) [21]. These differences might be due to the fact that postpartum women may not realize that they are at risk of pregnancy even if they are breastfeeding. On the other hand, the difference could be the fact that this study was done on women who were in the postpartum period when there was a high motivation to use family planning methods.

This study found that primary level education of postpartum women was significantly associated with modern contraceptive use. This may be explained as women who have been educated are more likely to visit a health facility and receive counseling or services on family planning, and go on to use modern contraceptives, than who have not been educated. Studies elsewhere have revealed a similar pattern of relationship between educational level and modern contraceptive use [22, 23].

Discussing modern FP methods with partner was significantly associated with postpartum modern contraceptive use. This finding is supported by reports from Kebri Beyah Ethiopia, Nairobi, rural Uganda [24–26]. This is because women who discuss modern FP with their partners are more likely to get acceptance and support since decision about its relevance and use can be made jointly. This can also be explained by the fact that any factor that influences the partner's attitude towards contraceptives would also affect women's use of postpartum contraceptives either negatively or positively.

Women whose menses returned after birth were more likely to use modern contraceptive than to women with amenorrhea. This is likely because women may be aware of their fertility returning when menses resume. Amenorrheic women would perceive themselves to be less likely to become pregnant by assuming that amenorrhea would protect against pregnancy irrespective of the postpartum period. This finding is supported by reports from Gondar, Nairobi, and a Demography Health Survey based-analysis from 17 developing countries [25, 27–29].

The result of the study showed that women in the postpartum period who ever heard about modern FP from health extension workers, friends, and other information sources utilized the method better than those who had not heard about modern. This is explained by the fact that the level of awareness determines the use of modern contraceptives. This further can be justified as having a universal knowledge of modern contraceptive methods helps postpartum women to have a better understanding of the available at health facilities and the benefits of fertility regulation. This is consistent with Kiramu District and a study done in Kathmandu university (94.3%) [17, 30].

Furthermore, women who contacted health professionals providing the service were more likely to use modern contraceptive in the postpartum period. A study conducted in Malawi supports this finding [23]. This may be due to client satisfaction; if clients perceive themselves as treated well and having received high-quality services, they tend to continue contraceptive use after delivery. Women stated that their reasons for not using modern contraceptives during the postpartum period were menses not returned (started) to use FP methods, fear of side effects, no prior exposure to modern FP, desire for more children, and husband disapproval. Similar reasons have been documented in studies conducted in Malawi, Gondar, and North Ethiopia [8, 23, 27].

## 5. Conclusion

In conclusion, postpartum modern contraceptive use was found to be low in the study area (20.7%). The factors associated with postpartum modern contraceptive use were maternal educational level (primary education level), discussing modern family planning with partner, menses returning since birth, ever heard about modern FP, and having contacted health professionals providing the service. Therefore, health professionals should work on improvements in women's educational status, making awareness of the women and counseling of their husbands about the use of postpartum contraception, when fertility returned and risky timing for becoming pregnant. Postpartum family planning should be integrated with other maternal health services like ANC and postnatal care. In addition, policy makers and program managers need to focus on improving male involvement in maternal health care issues especially on family planning service.

## Figures and Tables

**Table 1:** Sociodemographic information of respondents at Burie District, Amhara Region, North West Ethiopia, 2017 (*n* = 681).

S.no.		Variables	Frequency	Percent
1	Age of respondents (*n* = 681)	15-19	6	0.9
		20-24	88	12.9
		25-29	227	33.3
		30-34	201	29.5
		≥35	159	23.4

2	Marital status (*n* = 681)	Single	7	1.0
		Married	641	94.1
		Divorced	24	3.5
		Widowed	2	0.3
		Separated	7	1.0

3	Place of residence (*n* = 681)	Urban	95	14
		Rural	586	86

4	Religion (*n* = 681)	Orthodox	671	98.5
		Muslim	6	0.9
		Protestant	4	0.6

5	Ethnicity (*n* = 681)	Amhara	670	98.4
		Oromo	11	1.6

7	Educational status of husband (*n* = 641)	Cannot read and write	277	40.7
		Primary (1-8)	408	41.1
		Secondary (9-12)	67	9.8
		12 + 1 and above	17	2.5

8	Occupational status of women (*n* = 681)	Farmer	571	83.8
		Merchant	75	11
		Daily laborer	26	3.8
		Student	2	0.3
		Others	7	1.0

9	Occupational status of husband (*n* = 641)	Farmer	546	80.2
		Merchant	64	9.4
		Daily laborer	18	2.6
		Others	13	1.9

10	Monthly family income (*n* = 681)	<1,000	437	64.2
		1,000-1,500	122	17.9
		1,501-2,000	72	10.6
		>2,000	50	7.3

11	Media access (*n* = 681)	Yes	306	44.9
		No	375	55.1

	Types of media (*n* = 320) more than 1	Television	67	21
		Radio	253	79

12	Family size (*n* = 681)	2-3	159	23.3
		4-5	314	46.1
		5-6	162	23.8
		>6	46	6.8

**Table 2:** Pregnancy and child birth history of respondents at Burie District, Amhara Region, North West Ethiopia, 2017 (*n* = 681).

S.N	Variables		Frequency	Percent
1	No of pregnancies (*n* = 681)	1-2	304	44.6
		3-4	253	37.2
		5-6	101	14.8
		>6	23	3.4

2	No of live births (*n* = 681)	1-2	317	46.5
		3-4	256	37.6
		5-6	89	13.1
		>6	19	2.8

3	No of alive children (*n* = 681)	1-2	323	47.4
		3-4	254	37.3
		5-6	90	13.2
		>6	14	2.1

4	History of pregnancy within one year of birth before the index birth (*n* = 681)	Yes	49	7.2
		No	632	92.8

5	Reasons to become pregnant within one year before the index birth (*n* = 49)	Need of another child	11	22.45
		Husband disapproval to use modern FP	26	53.06
		Menses not returned to start modern FP	5	10.2
		Sex preference	7	14.29

6	Outcome of previous pregnancy (*n* = 49)	Terminated by medical abortion	14	28.58
		Terminated spontaneously	5	10.2
		Born alive	30	61.22

7	Current ANC follow up (*n* = 681)	Yes	67	9.8
		No	614	90.2

8	Status of the current pregnancy (*n* = 67)	Wanted	8	11.9
		Unwanted	24	35.8
		Planned	3	4.5
		Unplanned	32	47.8

9	Time in which pregnancy occurred after the index birth (*n* = 67)	<3 months	3	4.5
		3-6 months	2	3.0
		6-9 months	8	11.9
		9-12 months	54	80.6

10	Reasons to be pregnant currently (*n* = 67)	FP method failure	2	2.98
		Need of another child	10	14.91
		Husband disapproval to use modern FP	22	32.8
		Menses not returned to start modern FP	33	49.3

11	No of children you want to have in life (*n* = 681)	1	8	1.2
		2-3	82	12
		4-5	385	56.53
		6-7	191	28
		>7	15	2.2

**Table 3:** Factors affecting postpartum modern contraceptive use of respondents in Burie District, Amhara Region, North West Ethiopia, 2017 (*n* = 681).

Variables	Modern FP utilization use		COR (95% CI)	AOR (95% CI)
	Yes	No		
Women educational status				
Cannot read and write	66 (16.3%)	339(83.7%)		1.00
Primary (1-8)	57 (25.25%)	168 (74.75%)	0.139 [0.043-0.451]	0.15 [0.03-0.71]^∗^
Secondary (9-12)	11 (28.2%)	28 (71.8%)	0.242 [0.074-0.794]	0.281 [0.061-1.296]
12 + 1 and above	7 (58.3%)	5 (41.7%)	0.281 [0.073-1.075]	0.343 [0.067-1.744]
Total	141 (20.7%)	540 (79.3%)		
Discussion about modern FP				
Yes	80 (30%)	187 (70%)	0.389 [0.336-0.712]	0.60 [0.40-0.90]^∗^
No	61 (14.7%)	353 (85.3%)		1.00
Total	141 (20.7%)	540 (79.3%)		
Modern FP use before the index birth				
Yes	113 (23.3%)	372 (76.7%)	0.923 [0.718-3.038]	1.193 [0.692-2.055]
No	28 (14.3%)	168 (85.7%)		1.00
Total	141 (20.7%)	540 (79.3%)		
Menses return after birth				
Yes	86 (31.6%)	186 (68.4%)	0.354 [0.242-0.517]	0.39 [0.25-0.59]^∗^
No	55 (13.4)	354 (86.6%)		1.00
Total	141 (20.7%)	540 (79.3%)		
Occupational				
Farmer	113 (20.7%)	433 (79.3%)		1.00
Status of husband				
Merchant	22 (34.4%)	42 (65.6%)	2.030 [1.164-3.040]	3.118 [0.536-18.121]
Daily laborer	4 (22.2%)	14 (77.8%)	1.107 [0.357-3.429]	6.446 [0.07-11.78]^∗^
Others	2 (15.4%)	11 (84.6%)	0.705[.154-3.224]	8.769 [0.097-10.10]^∗^
Total	141 (22%)	500 (78%)		
Media access				
Yes	74 (24.25)	232 (75.8%)	1.466 [1.011-2.127]	0.933 [0.]
No	67 (17.8%)	308 (82.2%)		1.00
Total	141 (20.7%)	540 (79.3%)		
Occurrence preg. without menses return				
Yes	91 (31.7%)	196 (68.3%)	2.018 [1.387-2.936]	0.636 [0.369-1.097]
No	50 (12.7%)	344 (87.3%)		1.00
Total	141 (20.7%)	540 (79.3%)		
Ever heard about modern FP methods				
Yes	139 (23.3%)	457 (76.7%)	12.50 [3.037-51.492]	0.06 [0.01-0.43]^∗^
No	2 (2.4%)	83 (97.6%)		1.00
Total	141 (20.7%)	540 (79.3%)		
Health professor approach				
Agree	111 (25%)	332 (75%)	0.431 [0.278-0.669]	0.992 [0.537-1.831]
Disagree	30 (12.6%)	208 (87.4%)		1.00
Total	141 (20.7%)	540 (79.3%)		
Health professor available in				
Agree	99 (26.3%)	277 (73.7%)	2.238 [1.503-3.333]	1.85 [1.19-2.88]^∗^
Disagree	42 (13.8%)	263 (86.2%)		1.00

## Data Availability

The data used to support the findings of this study are available from the corresponding author upon request.

## References

[1] DaVanzo J., Hale L., Razzaque A., Rahman M. (2007). Effects of interpregnancy interval and outcome of the preceding pregnancy on pregnancy outcomes in Matlab, Bangladesh. *BJOG: An International Journal of Obstetrics & Gynaecology*.

[2] Jackson E., Glasier A. (2011). Return of ovulation and menses in postpartum Nonlactating Women. *Obstetrics & Gynecology*.

[3] Ross J. A., Winfrey W. L. (2001). Contraceptive use, intention to use and unmet need during the extended postpartum period. *International Family Planning Perspectives*.

[4] Vernon R. (2009). Meeting the family planning needs of postpartum women. *Studies in Family Planning*.

[5] WHO (2012). *Family Planning: A Health and Development Issue, A Key Intervention for the Survival of Women and Children*.

[6] World Health Organization (2013). *Programming Strategies for Postpartum Family Planning, Human Reproduction Program me*.

[7] Singh S., Darroch J. E. (2012). *Adding it up: Costs and benefits of contraceptive service*.

[8] Abraha T. H., Teferra A. S., Gelagay A. A. (2017). Postpartum modern contraceptive use in northern Ethiopia: prevalence and associated factors - methodological issue in this cross-sectional study. *Epidemiology and Health*.

[9] Pörtner C. C., Beegle K., Christiaensen L. (2011). *Family planning and fertility: estimating program effects using cross-sectional data*.

[10] Shonkoff J. P., Richter L., van der Gaag J., Bhutta Z. A. (2012). An integrated scientific framework for child survival and early childhood development. *Pediatrics*.

[11] (2015). *Best practice in postpartum family planning*.

[12] Philippines *Postpartum Family Planning Supplemented to Philippines Clinical Standards Manual on Family Planning*.

[13] FMOH (2015). *Health Sector Transformation Plan2015/16 - 2019/20(2008-2012 EFY)*.

[14] USAID (2011). *Family Planning Needs during the First Two Years Postpartum in Ethiopia*.

[15] Gaffield M. E., Egan S., Temmerman M. (2014). *It’s about Time: WHO and Partners Release Programming Strategies for Postpartum Family Planning*.

[16] Mengesha Z. B., Worku A. G., Feleke S. A. (2015). Contraceptive adoption in the extended postpartum period is low in Northwest Ethiopia. *BMC Pregnancy and Childbirth*.

[17] Kenate W., Amenu D. (2015). Assessment of contraceptive needs and practices of women during the extended postpartum period in Kiramu Woreda, Western Ethiopia. *International Journal of Advanced Biological and Biomedical Research*.

[18] Borda M., WilliamWinfery (2010). *Postpartum Fertility and Contraception: An Analysis of Finding from 17 Counties*.

[19] Tamrie Y. E., Hanna E. G., Argaw M. D. (2015). Determinants of long acting reversible contraception method use among mothers in extended postpartum period, Durame Town, Southern Ethiopia: a cross sectional community based survey. *Health*.

[20] Musa A., Assefa N., Weldegebreal F., Mitiku H., Teklemariam Z. (2016). Factor associated with experience of modern contraceptive use before pregnancy among women who gave birth in Kersa HDSS, Ethiopia. *BMC Public Health*.

[21] Do M., Hotchkiss D. (2013). Relationships between antenatal and postnatal care and post-partum modern contraceptive use: evidence from population surveys in Kenya and Zambia. *BMC Health Services Research*.

[22] Tawfeek R. S., Khaleel H. A., Mustafa Z. M. (2011). Spacing Effects on Maternal-Child Health. A Hospital Based Study at Tikrit Teaching Hospital. *Tikrit Medical Journal*.

[23] Bwazi C., Maluwa A., Chimwaza A., Pindani M. (2014). Utilization of postpartum family planning services between six and twelve months of delivery at Ntchisi District Hospital, Malawi. *Health*.

[24] Nigussie A. T., Girma D., Tura G. (2016). Postpartum family planning utilization and associated factors among women who gave birth in the past 12 months, Kebribeyah town, Somali region, eastern Ethiopia. *Journal of Women's Health Care*.

[25] Ndugwa R. P., Cleland J., Madise N. J., Fotso J.-C., Zulu E. M. (2011). Menstrual pattern, sexual behaviors, and contraceptive use among postpartum women in Nairobi urban slums. *Journal of Urban Health*.

[26] Rutaremwa G., Kabagenyi A., Wandera S. O., Jhamba T., Akiror E., Nviiri H. L. (2015). Predictors of modern contraceptive use during the postpartum period among women in Uganda: a population-based cross sectional study. *BMC Public Health*.

[27] Abera Y., Mengesha Z. B., Tessema G. A. (2015). Postpartum contraceptive use in Gondar town, Northwest Ethiopia: a community based cross-sectional study. *BMC Women's Health*.

[28] Sarvamangala K., Taranum A. (2013). Menstrual pattern, sexual behavior and contraceptive use among postpartum women in a tertiary care hospital in Davangere. *Medica Innovatica*.

[29] Borda M. R., Winfrey W., McKaig C. (2010). Return to sexual activity and modern family planning use in the extended postpartum period: an analysis of findings from seventeen countries. *African Journal of Reproductive Health*.

[30] Haile A., Enqueselassie F. (2009). Influence of women's autonomy on couple's contraception use in Jimma town, Ethiopia. *Ethiopian Journal of Health Development*.

